# Elevated ALT/AST ratio as a marker for NAFLD risk and severity: insights from a cross-sectional analysis in the United States

**DOI:** 10.3389/fendo.2024.1457598

**Published:** 2024-08-26

**Authors:** Yanyan Xuan, Dingting Wu, Qin Zhang, Zhiqiang Yu, Jingbo Yu, Dongdong Zhou

**Affiliations:** ^1^ Department of Hospital Infection, The First Affiliated Hospital of Ningbo University, Ningbo, Zhejiang, China; ^2^ Department of Hepatology, The First Affiliated Hospital of Ningbo University, Ningbo, Zhejiang, China; ^3^ Department of General Practice, The First Affiliated Hospital of Ningbo University, Ningbo, Zhejiang, China; ^4^ Department of Clinical Nutrition, Sir Run Run Shaw Hospital, Zhejiang University School of Medicine, Hangzhou, Zhejiang, China; ^5^ Electronic Information School, Zhejiang Business Technology Institute, Ningbo, Zhejiang, China; ^6^ Department of Geriatrics Medicine, The First Affiliated Hospital of Ningbo University, Ningbo, Zhejiang, China

**Keywords:** NAFLD, alanine aminotransferase, aspartate aminotransferase, NHANES, steatosis, fibrosis

## Abstract

**Background:**

The prevalence and incidence of Nonalcoholic fatty liver disease (NAFLD) are increasing worldwide, and NAFLD has emerged as a prominent global health concern. The link between serum alanine aminotransferase (ALT) to aspartate aminotransferase (AST) ratio and NAFLD remains unclear. This study investigated the association between the ALT/AST ratio and NAFLD prevalence, including liver steatosis and fibrosis levels in the population.

**Methods:**

We conducted a cross-sectional study using data from the National Health and Nutrition Examination Survey (NHANES) 2017–2018, including 4753 participants. Subgroup analyses, stratified by age, gender, and body mass index (BMI), were performed, along with adjusted multivariable logistic regression analyses to evaluate the relationship between ALT/AST levels and the likelihood of NAFLD, liver steatosis, and hepatic fibrosis stage. A generalized additive model examined the non-linear relationship between ALT/AST and the probability of developing NAFLD.

**Results:**

Among 4753 participants, 1508 (31.73%) were diagnosed with NAFLD. Significant positive correlations between ALT/AST and NAFLD risk were found across all models. In addition, the subgroup analysis by gender, age, and BMI suggested that ALT/AST showed a positive correlation with NAFLD. The ALT/AST ratio was positively correlated with the degree of liver steatosis and liver fibrosis. The correlation between ALT/AST and the incidence of NAFLD showed a non-linear pattern. In women, the non-linear trend is particularly evident, showing an inverted U-shaped curve with an inflection point of 1.302. A receiver operating characteristic (ROC) analysis showed that the predictive value of ALT/AST for NAFLD was better than that of traditional liver enzyme parameters.

**Conclusion:**

A higher ALT/AST ratio was independently associated with a significantly higher risk of NAFLD and liver fibrosis within American cohorts. This link is robust among females, children, and adolescents. ALT/AST ratio can be used as a simple and effective noninvasive biomarker to identify individuals with high risk of NAFLD.

## 1 Introduction

Nonalcoholic fatty liver disease (NAFLD) is defined as a pathological feature of hepatocellular steatosis (steatosis present in more than 5% of liver cells), in which the patient has no history of excessive alcohol consumption and other liver damage caused by viral infection, drugs or autoimmune disease is excluded (1, 2). NAFLD is currently the most common chronic liver disease worldwide and the leading cause of elevated serum transaminases in healthy people undergoing physical examination. NAFLD has become an emerging and significant public health problem (3). It is now estimated that 32.4% of people worldwide have NAFLD. The prevalence is higher in men than women and is increasing every year, with Latin America having the highest prevalence (44.4%) and Western Europe the lowest (25.1%) (4). Nonalcoholic steatohepatitis(NASH) has emerged as the second most prevalent cause of liver disease among individuals receiving liver transplantation in the United States, and fatty liver disease is now the most common liver disease in the country and other Western nations (5, 6). Patients with NAFLD have a poor prognosis, mainly due to cardiovascular disease and non-liver malignancies, which together are eight times more common than hepatocellular carcinoma (7, 8). Additionally, individuals with NAFLD have an increased risk of developing cardiovascular and cerebrovascular illnesses. In comparison to healthy individuals, NAFLD patients have far higher incidence rates of diabetes, hypertension, coronary heart disease, and chronic kidney disease (9, 10).

Asymptomatic elevation of liver enzymes is a possible clinical manifestation of NAFLD. Of these, alanine aminotransferase (ALT) is most closely associated with fat accumulation in the liver and is most commonly found in the cytoplasm of hepatocytes. Serum ALT has historically been utilized as a measure of inflammation for liver cell damage in conditions including cirrhosis, hepatitis, liver cancer, and alcoholic liver disease (11–13). As ALT is a sensitive indicator for identifying NAFLD damage, it frequently rises when liver damage is moderate. Even when ALT is within the normal range, NAFLD can still affect it (14, 15). In individuals with NAFLD, ALT is often greater than aspartate aminotransferase (AST), and AST/ALT<1 is the typical presentation. Cells’ cytoplasm and mitochondria contain AST, and liver cells release more AST than ALT as the disease progresses because the cells’ mitochondria are damaged. The increase in ALT and AST varies with the degree and duration of liver disease, so the ALT/AST ratio has considerable clinical significance in diagnosing liver disease (16, 17). Fernando De Ritis first proposed the ALT/AST ratio in 1957 to measure the severity of viral hepatitis (18). ALT/AST has since been utilized to assist in the diagnosis of cancer, diabetes, chronic renal disease, cardiovascular disease, and liver disease (19–21). Recent research has demonstrated a significant correlation between insulin resistance and liver enzymes, and ALT/AST is considered a reliable biomarker of insulin resistance in Asian populations (20, 22–24). In an earlier study called the Framingham Steatosis Index, which was designed to identify patients with liver steatosis, the ALT/AST ratio was found to be related to the amount of liver fat in liver biopsies. The ALT/AST ratio, especially when the aminotransferase result is within the normal range, may be a more reliable diagnostic of hepatic steatosis than AST or ALT alone (25).

Few studies evaluate the ALT/AST ratio as a marker for NAFLD, and none have examined the association between the ALT/AST ratio and NAFLD in the general American population. Therefore, the specific objective of this study is to explore the relationship between the ALT/AST ratio and the degree of liver steatosis, and the progression of fibrosis in the general US population. Concurrently, a subgroup analysis based on gender, age, and BMI stratification was performed further to evaluate the relationship between ALT/AST and NAFLD.

## 2 Methods

### 2.1 Study design and population

The National Center for Health Statistics (NCHS) developed and conducted the National Health and Nutrition Examination Survey (NHANES) to address emerging public health issues and to provide unbiased information on diseases affecting adults and children in the United States. A massive, continuous, cross-sectional survey with a nationally representative sample is called NHANES. Researchers around the world have unrestricted access to NHANES data via the Internet. Each survey participant undergoes a thorough assessment process, including a home interview and a mobile examination center (MEC) evaluation that includes specialized tests, laboratory work, and a physical examination. The data can be viewed at https://www.cdc.gov/nch s/nhanes.htm, and detailed descriptions of the NHANES methodology can be found here. The NCHS Research Ethics Review Board approved the procedure for the NHANES survey, and each member completed a written consent form.

**Figure 1:**
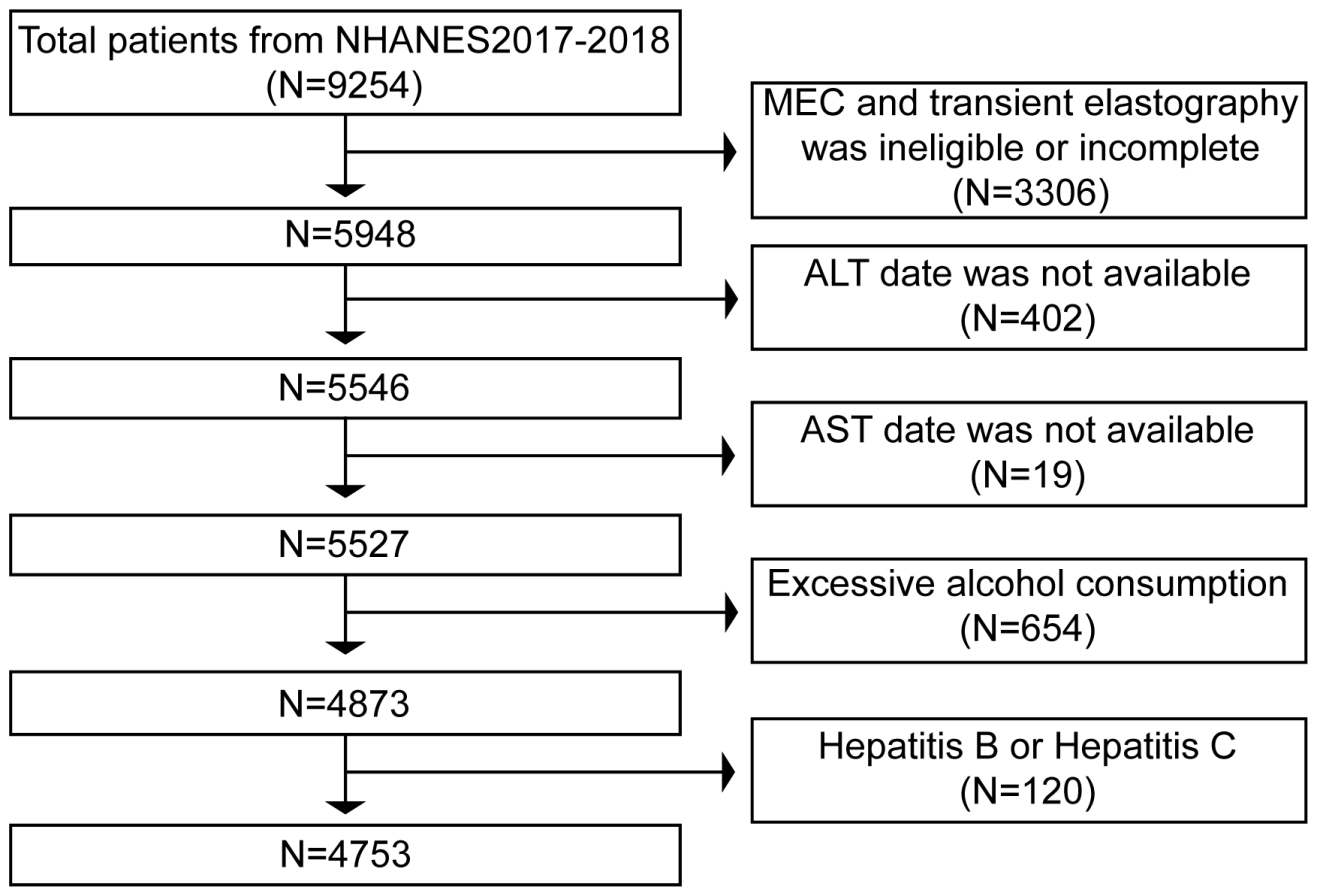
Flowchart of the study participants.

The liver ultrasonography transient elastography (TE) examination employed in this analysis, for which data were gathered from the NHANES 2017–2018, was mainly based on the diagnosis of NAFLD. 9,254 people took part in the study, and 3,306 people with incomplete MEC exams or no TE data were not included in the analysis. Then, the 402 individuals for whom ALT statistics were lacking and the 19 individuals whose AST values were unavailable were eliminated. Finally, we remove 654 individuals who consume excessive amounts of alcohol, defined as more than three drinks per day for men and more than two drinks per day for women. We also remove 50 individuals with hepatitis B (hepatitis B surface antigen positive) and 70 with hepatitis C (hepatitis C antibody positive). Finally, the current study included 4,753 individuals (**Figure 1**). The STROBE (Strengthening the Reporting of Observational Studies in Epidemiology) technique was used to write this report.

### 2.2 Definition of NAFLD

NAFLD has been assessed using vibration-activated transient elastography (VCTE) data with a controlled attenuation parameter (CAP). CAP values vary from 100 to 400 dB/m, with higher values corresponding to more significant amounts of liver fat. Based on data from a previous population-based meta-analysis that looked at CAP diagnostic cutoffs for the disease, NAFLD had a CAP score of at least 288 dB/m (6).

### 2.3 Vibration controlled transient elastography

Liver biopsy is now the gold standard for determining the degree of liver steatosis and identifying NAFLD. However, liver biopsy has several drawbacks, including high cost and poor reproducibility, as well as bleeding, infection, and even death (1: 10,000) (26, 27). As a noninvasive method to assess the incidence and severity of NAFLD, physicians often use VCTE in clinical practice. Using the CAP and Liver Stiffness Measurement (LSM) is highly accurate in detecting the presence of both liver steatosis and fibrosis (28).

During the 2017-2018 cycle, the FibroScan® model 502 V2 Touch was used to measure VCTE using a medium (M) and extra-large (XL) probe. According to previous clinical evidence, the degree of liver fibrosis and steatosis was strongly associated with liver LSM and CAP levels (29). Decibels per meter (dB/m) and kilopascals (kPa) were used to denote the values for CAP and LSM, respectively. For this study, tests were considered credible if at least 10 LSM values with an interquartile range/median (IQRe) of less than 30% were obtained after a minimum 3-hour fast. A previous study found that the cutoff value for hepatic steatosis as assessed by VCTE using CAP was greater than or equal to 288 dB/m) (6). In addition, significant liver fibrosis (F2), advanced liver fibrosis (F3), and cirrhosis (F4) are indicated by LSM values of 8.0, 9.7, and 13.7 kPa, respectively (30).

### 2.4 Variables

Hepatic steatosis severity, liver fibrosis level, and NAFLD prevalence were dependent variables, while ALT/AST was the independent variable. Confounders that have been shown via prior research and clinical practice were used as covariates in this study. Our study included sex, race, smoking status, level of physical activity, hypertension status, and Type 2 Diabetes (T2DM) as categorical variables used as covariates. In our study, we further used the subsequent continuous variables as covariates: Gamma-glutamyl transpeptidase (GGT), alkaline phosphatase (ALP), Total bilirubin (TBIL), serum lipids, serum uric acid (SUA), C-reactive protein (CRP), platelet (PLT), fast glucose, fast insulin, glycated hemoglobin (HbA1c), systolic/diastolic blood pressure (S/DBP), and Body mass index (BMI).

The diagnosis of T2DM was made using the following criteria, which were derived from the American Diabetes Association: First, self-reported diabetes; second, usage of anti-diabetic drugs; third, measurement of 126 mg/dl (7 mmol/L) or higher for fasting plasma glucose (FPG); fourth, measurement of ≥ 200 mg/dl (11.1 mmol/L) for random plasma glucose; and fifth, measurement of ≥ 6.5% for HbA1c (31). Hypertension was defined as (1) A systolic blood pressure of more than 140 mmHg or diastolic blood pressure of more than 90 mmHg was recorded; (2) Antihypertensive medications are currently being used; and (3) Self-reported hypertension (32). If any of these conditions were met, it can be diagnosed as hypertension. Testing for the hepatitis B surface antigen detects HBV, while HCV infection is confirmed by testing positive for HCV antibodies or by the presence of HCV RNA. The weight in kilograms divided by the height in square meters was the formula for determining BMI. In addition, the requirements of the physical activity guidelines were followed to classify the activity level as active, moderate, or inactive.

### 2.5 Statistical analysis

The data analysis in this study took into account sampling weights based on the analytical guideline designed by NCHS and was conducted using package R version 3.4.3 (http://www.R-project.org) and EmpowerStats software (http://www.empowerstats.com). The data analysis used the proper weighting that the NCHS suggested to guarantee that the results appropriately reflect the US population. A two-tailed P value of less than 0.05 was needed to establish statistical significance. Continuous variables were described as a weighted mean and standard deviation if normally distributed or otherwise as the median and interquartile range (IQR). Categorical variables were expressed as weighted frequencies and proportions. Three types of logistic regression models were created once ALT/AST was categorized quarterly to investigate the association between ALT/AST and NAFLD as well as liver fibrosis: (1) No covariates have been adjusted for; (2) age, gender, and race adjustments have been made; (3) all pertinent variables, including age, gender, race, hypertension, BMI, physical activity level, T2DM, smoke, waist circumference (WC), LSM, DBP, SBP, HbA1C, CRP, PLT, fast glucose, fast insulin, TBIL, alkaline phosphatase (ALP), Gamma-glutamyl transpeptidase (GGT), total cholesterol (TC), Triglycerides (TG), and SUA, have been adjusted for.

We also performed subgroup stratification by sex, age, or BMI to identify appropriate groups using categorized multivariate logistic regression. In addition, threshold effect analysis and smooth curve fitting were used to investigate further the non-linear relationship between ALT/AST ratio and NAFLD. Binary linear regression models were performed on both sides of the threshold point after identifying non-linearities. Receiver operator characteristic (ROC) curve studies were conducted to assess the effectiveness of ALT/AST or other biochemical markers in identifying NAFLD. The aim was to determine the optimal ALT/AST ratio for calculating NAFLD risk in this population. The best cutoffs were found by maximizing the Yoden index. The result was considered statistically significant if the p-value was less than 0.05.

## 3 Results

### 3.1 Demographic and clinical characteristics of the study population

4753 people took part in the survey. Participants’ average age was 43.27 ± 19.33 years, and 54.43% identified as female and 45.57% as male. The weighted distributions of characteristics by whether they were NAFLD (**Table 1**).

**Table 1:** Weighted characteristics of participants with and without NAFLD status.

Characteristics	Non-NAFLD (n = 3245)	NAFLD (n=1508)	P value
Age (years)	40.64 ± 19.73	49.02 ± 17.06	< 0.0001
Age (%)			< 0.0001
<20	17.63	4.84	
≥ 20, <40	34.97	25.73	
≥ 40, <60	26.58	39.93	
≥ 60	20.82	29.46	
Gender (%)			< 0.0001
Male	41.83	53.58	
Female	58.18	46.42	
Race (%)			< 0.0001
Mexican American	8.51	13.98	
Other Hispanic	7.47	6.06	
Non-Hispanic White	60.58	60.52	
Non-Hispanic Black	12.08	8.38	
Non-Hispanic Asian	6.27	5.55	
Other Race	5.08	4.51	
Smoking behavior (%)			< 0.0001
Never smoke	71.51	60.43	
Ever smoke	17.57	26.38	
Current smoke	10.92	13.19	
Hypertension (%)			< 0.0001
No	72.19	43.45	
Yes	27.81	56.56	
T2DM			< 0.0001
No	93.35	74.10	
Yes	6.65	25.90	
Physical activity level			0.1204
inactive	49.57	47.01	
moderate	8.62	10.25	
active	41.81	42.74	
BMI (Kg/m^2^)	26.61 ± 6.06	34.35 ± 7.40	< 0.0001
BMI (%)			< 0.0001
< 25	43.86	5.10	
≥ 25, <30	31.84	25.36	
≥ 30	24.30	69.54	
WC (cm)	91.36 ± 15.24	112.20 ± 15.58	< 0.0001
HC (cm)	102.08 ± 12.33	115.62 ± 14.86	< 0.0001
SBP	118.38 ± 17.41	127.02 ± 16.90	< 0.0001
DBP	69.88 ± 12.05	74.23 ± 12.13	< 0.0001
PLT (10^9^/L)	245.61 ± 59.79	253.73 ± 62.89	< 0.0001
CRP (mg/L)	2.88 ± 6.58	4.99 ± 7.74	< 0.0001
Fast glucose (mmol/L)	5.15 ± 1.05	6.03 ± 2.20	< 0.0001
Fast insulin (mIU/L)	9.85 ± 9.05	18.91 ± 15.17	< 0.0001
Glycohemoglobin (%)	5.43 ± 0.63	5.99 ± 1.10	< 0.0001
ALT (IU/L)	18.71 ± 13.37	27.89 ± 19.49	< 0.0001
GGT (IU/L)	21.60 ± 24.19	34.97 ± 37.61	< 0.0001
AST (IU/L)	20.51 ± 10.33	23.06 ± 13.11	< 0.0001
ALP (IU/L)	87.00 ± 55.34	82.45 ± 31.55	0.0031
TBIL (mg/dL)	0.48 ± 0.30	0.44 ± 0.25	< 0.0001
TG (mg/dL)	113.42 ± 69.78	179.67 ± 121.51	< 0.0001
TC (mg/dL)	181.86 ± 39.30	190.12 ± 40.47	< 0.0001
HDL-C (mg/dL)	56.43 ± 14.57	47.68 ± 13.04	< 0.0001
LDL-C (mg/dL)	105.43 ± 33.69	113.24 ± 36.35	< 0.0001
SUA (mg/dL)	5.05 ± 1.32	5.79 ± 1.41	< 0.0001
ALT/AST	0.90 ± 0.32	1.18 ± 0.38	< 0.0001
CAP (dB/m)	222.85 ± 40.36	322.00 ± 32.83	< 0.0001
LSM (kPa)	4.90 ± 2.88	7.30 ± 7.04	< 0.0001

Mean ± SD was for continuous variables. The weighted linear regression model calculated the p-value. % was for categorical variables. The weighted chi-square test calculated the p-value. NAFLD, Non-alcoholic fatty liver disease; T2DM, Type 2 diabetes; BMI, Body mass index; SBP, Systolic blood pressure; DBP, Diastolic blood pressure; PLT, Platelet; CRP, C-Reactive Protein; ALT, Alanine aminotransferase; AST, Aspartate aminotransferase; GGT, Gamma-glutamyl transpeptidase; TBIL, Total bilirubin; HbA1c, Glycosylated hemoglobin A1c; TG, Triglycerides; TC, Total cholesterol; HDL-C, High-density lipoprotein cholesterol; LDL-C, Low-density lipoprotein cholesterol; SUA, Serum uric acid; LSM, Liver stiffness measurement; CAP, Controlled attenuation parameter.

NAFLD participants had higher rates of smoking, hypertension, T2DM, older, male, and Mexican-American. Significantly lower levels of HDL-C and TBIL were found in people with NAFLD, but much higher levels of BMI, WC, hip circumference (HC), S/DBP, LSM, CAP, TG, fast glucose, TC, fast insulin, low-density lipoprotein cholesterol (LDL-C), HbA1c, PLT, ALT, AST, CRP, GGT, and SUA (*P* < 0.001 for each). In addition, ALT/AST was significantly lower in the non-NAFLD group than in the NAFLD subgroup (0.90 ± 0.32 *vs*. 1.18 ± 0.38, *P* < 0.0001). However, there were no significant differences in physical activity levels.

### 3.2 Correlation between NAFLD and ALT/AST

Multiple regression analysis was used to examine the relationship between the ALT/AST ratio and the prevalence of NAFLD (**Table 2**). All three multivariable logistic regression models showed positive correlations between ALT/AST and the risk of NAFLD: model 1 (OR = 9.201, 95% CI: 7.536, 11.234), model 2 (OR = 9.732, 95% CI: 7.853, 12.060), and model 3 (OR = 3.648, 95% CI: 2.827, 4.706). When ALT/AST ratio was converted from a continuous variable to a categorical variable (quartiles), participants in quartile 2 (ALT/AST 0.708-0.881), quartile 3 (ALT/AST 0.882-1.132) and quartile 4 (ALT/AST 1.133-3.314) were associated with 6.6%, 83%, 215.4% higher odds of being NAFLD, respectively, compared with quartile1 (ALT/AST 0.167-0.707). The findings indicated that NAFLD was more likely to develop in individuals with higher ALT/AST than those with lower ALT/AST.

**Table 2:** Associations between ALT/AST and NAFLD status.

	Model 1 *OR* (95% CI), *P* value	Model 2 *OR* (95% CI), *P* value	Model 3 *OR* (95% CI), *P* value
**ALT/AST**	9.201 (7.536, 11.234) < 0.001	9.732 (7.853, 12.060) < 0.001	3.648 (2.827, 4.706) < 0.001
Q1 (0.167-0.707)	Reference	Reference	Reference
Q2 (0.708-0.881)	1.739 (1.396, 2.167) < 0.001	1.520 (1.212, 1.905) < 0.001	1.066 (0.817, 1.390) 0.6385
Q3 (0.882-1.132)	3.838 (3.124, 4.716) < 0.001	3.278 (2.649, 4.055) < 0.001	1.830 (1.424, 2.352) < 0.001
Q4 (1.133-3.314)	8.588 (7.003, 10.531) < 0.001	8.017 (6.464, 9.944) < 0.001	3.154 (2.432, 4.090) < 0.001
*P* for trend	< 0.001	< 0.001	< 0.001
Subgroup analysis stratified by sex			
**Men**	7.746 (5.972, 10.047) < 0.001	8.960 (6.784, 11.833) < 0.001	3.030 (2.136, 4.299) < 0.001
Q1 (0.200-0.707)	Reference	Reference	Reference
Q2 (0.708-0.881)	1.717 (1.207, 2.443) 0.0026	1.337 (0.927, 1.930) 0.1205	0.751 (0.477, 1.181) 0.2147
Q3 (0.882-1.132)	3.357 (2.429, 4.638) < 0.001	2.564 (1.830, 3.592) < 0.001	1.267 (0.835, 1.922) 0.2652
Q4 (1.133-3.043)	8.299 (6.107, 11.278) < 0.001	7.477 (5.429, 10.296) < 0.001	2.108 (1.392, 3.193) < 0.001
*P* for trend	< 0.001	< 0.001	< 0.001
**Women**	11.364 (8.169, 15.808) < 0.001	11.224 (7.988, 15.771) < 0.001	4.189 (2.824, 6.214) < 0.001
Q1 (0.167-0.707)	Reference	Reference	Reference
Q2 (0.708-0.881)	1.750 (1.321, 2.319) < 0.001	1.652 (1.238, 2.205) < 0.001	1.211 (0.870, 1.685) 0.2573
Q3 (0.882-1.132)	4.186 (3.203, 5.471) < 0.001	3.865 (2.935, 5.090) < 0.001	2.255 (1.642, 3.097) < 0.001
Q4 (1.133-3.314)	8.077 (6.058, 10.768) < 0.001	7.903 (5.865, 10.648) < 0.001	3.719 (2.624, 5.272) < 0.001
*P* for trend	< 0.001	< 0.001	< 0.001
Subgroup analysis stratified by age			
< 20	22.528 (12.882, 39.399) < 0.001	22.792 (12.680, 40.970) < 0.001	7.570 (3.515, 16.306) < 0.001
≥ 20, <40	8.791 (6.137, 12.594) < 0.001	7.208 (4.855, 10.701) < 0.001	2.574 (1.578, 4.198) < 0.001
≥ 40, <60	7.299 (4.975, 10.709) < 0.001	6.721 (4.466, 10.115) < 0.001	3.270 (2.021, 5.291) < 0.001
≥ 60	8.592 (5.558, 13.281) < 0.001	7.868 (5.036, 12.292) < 0.001	3.999 (2.355, 6.791) < 0.001
Subgroup analysis stratified by BMI			
< 25	5.245 (2.938, 9.363) < 0.001	4.871 (2.573, 9.220) < 0.001	3.779 (1.810, 7.892) < 0.001
≥ 25, <30	3.996 (2.836, 5.631) < 0.001	3.840 (2.634, 5.599) < 0.001	3.500 (2.278, 5.379) < 0.001
≥ 30	5.429 (4.000, 7.368) < 0.001	4.641 (3.318, 6.493) < 0.001	3.322 (2.297, 4.805) < 0.001

Model 1: no covariates were adjusted. Model 2: age, gender, and race were adjusted. Model 3: age, gender, race, hypertension, BMI, T2DM, smoke, physical activity level, WC, LSM, DBP, SBP, CRP, fast glucose, fast insulin, HbA1c, TBIL, ALP, GGT, TC, TG, and SUA were adjusted. In the subgroup analysis for gender, the model was not adjusted for gender; in the subgroup analysis for age, the model was not adjusted for age; in the subgroup analysis for BMI, the model was not adjusted for BMI.

Gender-stratified subgroup analyses for all three models showed that male NAFLD risk and ALT/AST were positively correlated in model 1 (OR = 7.746, 95% CI: 5.972, 10.047), model 2 (OR = 8.960, 95% CI: 6.784, 11.833), and model 3 (OR = 3.030, 95% CI: 2.136, 4.299). Similar results were found in women, as all three models (OR = 11.364, 95% CI: 78.169, 15.808), model 2 (OR = 11.224, 95% CI: 7.988, 15.771), and model 3 (OR = 4.189, 95% CI: 2.824, 6.214) showed a positive association.

According to the subgroup analysis stratified by age, individuals younger than 20 years with higher ALT/AST were also more likely to develop NAFLD than those in other age groups: model 1 (OR = 22.528, 95% CI: 12.882, 39.399), model 2 (OR = 22.792, 95% CI: 12.680, 40.970) and model 3 (OR = 7.570, 95% CI: 3.515, 16.306).

All BMI groups showed a positive correlation with the prevalence of NAFLD; additionally, those with a BMI < 25 Kg/m^2^ had a greater risk of developing NAFLD than those in other groups: model 1 (OR = 5.245, 95% CI: 2.938, 9.363), model 2 (OR = 4.871, 95% CI: 2.573, 9.220), and model 3 (OR = 3.779, 95% CI: 1.810, 7.892).

### 3.3 Correlation between ALT/AST and the severity of hepatic steatosis

The association between ALT/AST and hepatic steatosis is based on CAP levels (**Supplementary Table 1**). It was demonstrated that ALT/AST was significantly and positively correlated with the degree of hepatic steatosis in models 1 (β = 70.570, 95% CI: 65.916, 75.224), 2 (β = 67.137, 95% CI: 62.499, 71.774) and 3 (β = 31.551, 95% CI: 26.806, 36.296), while the *P* for trend was less than 0.001. Moreover, the higher ALT/AST quartile had a significantly higher degree of hepatic steatosis than the lowest quartile (*P* for trend < 0.001). The degree of hepatic steatosis and ALT/AST were positively correlated in both males (β = 30.066, 95% CI: 23.639, 36.494, *P* < 0.001) and females (β = 29.812, 95% CI: 22.529, 37.094, *P* < 0.001) after adjustment for all relevant factors. Concurrently, subgroup analysis by age and BMI showed a high correlation between the degree of hepatic steatosis and ALT/AST, particularly in the group under 20 years or with a BMI between 25 and 30 Kg/m^2^.

### 3.4 Association between ALT/AST and the severity of liver fibrosis

The relationship between ALT/AST and the three stages of liver fibrosis (**Table 3**). According to our research, ALT/AST was associated with cirrhosis, advanced liver fibrosis, and significant liver fibrosis in both model 1 and model 2 (*P* < 0.01). After adjustment for all relevant factors, ALT/AST was associated with significant liver fibrosis model 3 (OR = 1.827, 95% CI: 1.354, 2.466) and advanced liver fibrosis model 3 (OR = 1.739, 95% CI: 1.197, 2.526).

**Table 3:** Association between ALT/AST and risk of hepatic fibrosis.

Degree of hepatic fibrosis	Model 1 OR (95% CI), P value	Model 2 OR (95% CI), P value	Model 3 OR (95% CI), P value
**Significant fibrosis** **(F2, LSM ≥ 8.0 kPa)**	2.215 (1.744, 2.813) < 0.001	2.503 (1.919, 3.265) < 0.001	1.827 (1.354, 2.466) < 0.001
**Men**	1.570 (1.145, 2.155) 0.0051	1.875 (1.328, 2.647) < 0.001	1.609 (1.089, 2.377) 0.01703
**Women**	3.573 (2.388, 5.345) < 0.001	3.980 (2.610, 6.068) < 0.001	2.498 (1.547, 4.033) < 0.001
**Advanced fibrosis** **(F3, LSM ≥ 9.7 kPa)**	2.103 (1.569, 2.817) < 0.001	2.361 (1.701, 3.278) < 0.001	1.739 (1.197, 2.526) 0.00371
**Men**	1.601 (1.090, 2.352) 0.0163	1.976 (1.291, 3.025) 0.0017	1.891 (1.166, 3.067) 0.0098
**Women**	2.930 (1.796, 4.780) < 0.001	3.201 (1.913, 5.358) < 0.001	1.830 (0.993, 3.372) 0.0525
**Cirrhosis** **(F4, LSM ≥ 13.7 kPa)**	1.844 (1.190, 2.858) 0.0062	1.914 (1.171, 3.127) 0.0095	1.160 (0.652, 2.064) 0.6133
**Men**	1.246 (0.697, 2.230) 0.4579	1.484 (0.781, 2.823) 0.2284	1.202 (0.565, 2.561) 0.6326
**Women**	2.915 (1.440, 5.902) 0.0029	3.035 (1.443, 6.380) 0.0034	1.568 (0.639, 3.850) 0.3264

Model 1: no covariates were adjusted. Model 2: age, gender, and race were adjusted. Model 3: age, gender, race, hypertension, BMI, T2DM, smoke, physical activity level, CAP, fast glucose, fast insulin, HbA1c, TBIL, ALP, GGT, TC, TG, CRP, and SUA were adjusted. In the subgroup analysis for gender, the model was not adjusted for gender.

According to all three multivariable logistic regression models, there was a positive connection (*P* < 0.05) between men’s ALT/AST and significant fibrosis and advanced fibrosis, according to subgroup analysis stratified by gender. The findings for the advanced liver fibrosis model 3 (OR = 1.891, 95% CI: 1.166, 3.067) and significant liver fibrosis model 3 (OR = 1.609, 95% CI: 1.089, 2.377) were as follows after adjusting for every covariate. Nevertheless, after controlling for all possible variables in model 3, we only discover an association between ALT/AST and significant liver fibrosis; we do not find an association with advanced liver fibrosis or cirrhosis in females (**Table 3**).

### 3.5 The analysis of non-linear relationship

**Figure 2:**
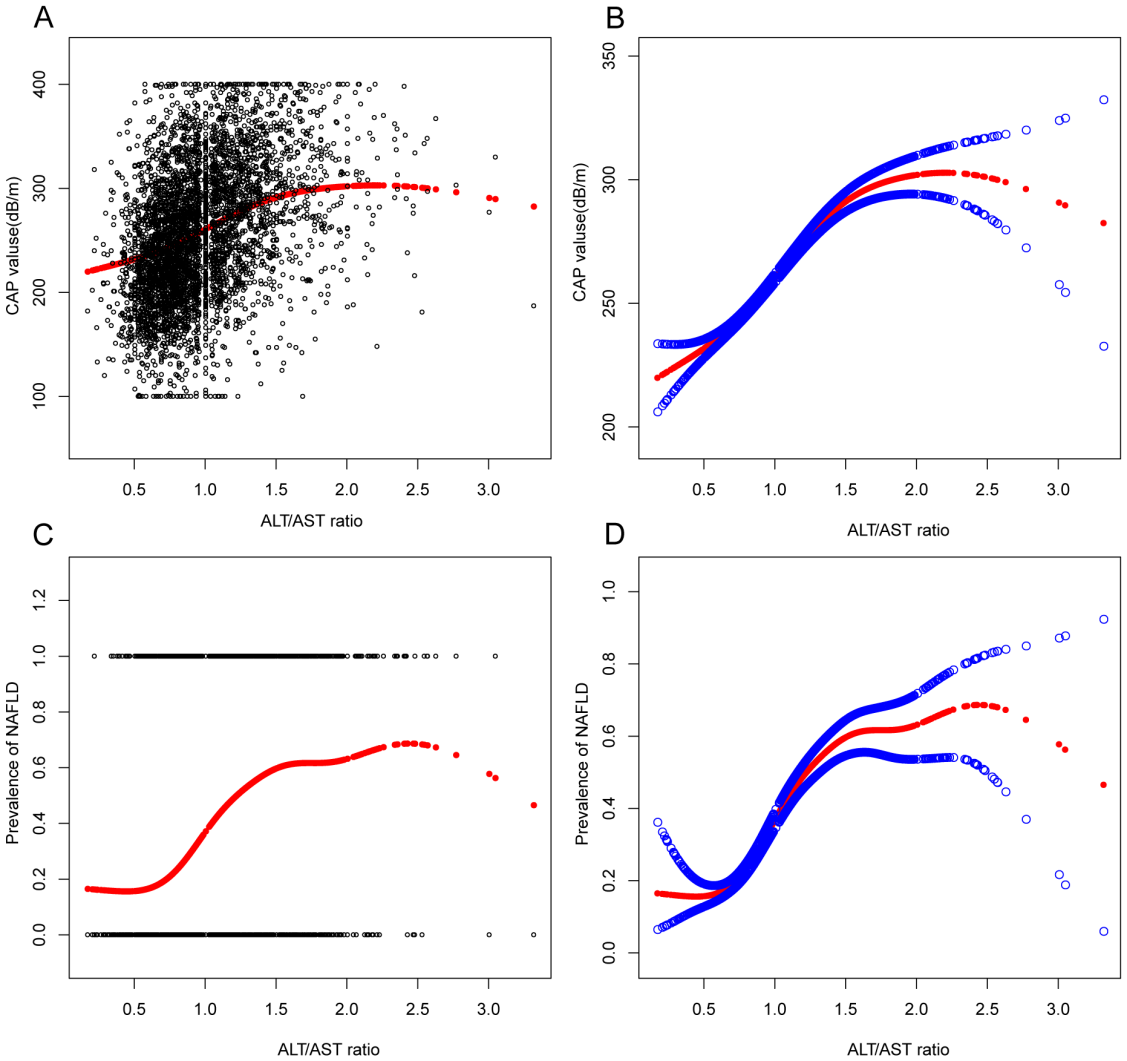
Associations between alanine aminotransferase to aspartate aminotransferase ratio and CAP values or prevalence of NAFLD. **(A, B)**: Associations between ALT/AST ratio and CAP values. **(C, D)**: Associations between ALT/AST ratio and prevalence of NAFLD. Each black point represents a sample. The solid red line represents the smooth curve fit between variables. Blue bands represent the 95% confidence interval from the fit. They were adjusted for age, gender, race, hypertension, BMI, T2DM, smoke, WC, LSM, DBP, SBP, CRP, fast glucose, fast insulin, HbA1c, TBIL, ALP, GGT, TC, TG, and SUA.

**Figure 3:**
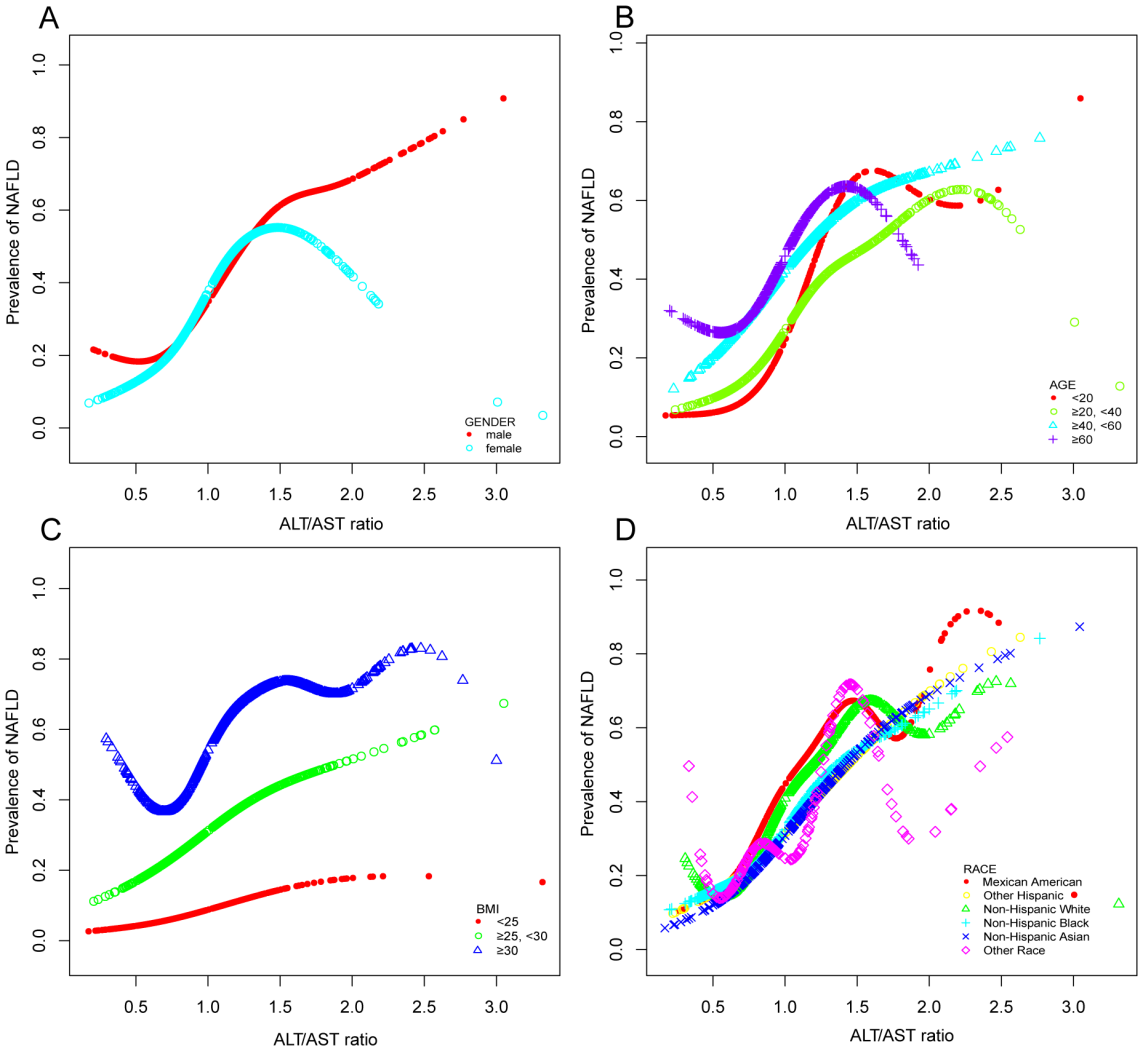
Associations between alanine aminotransferase to aspartate aminotransferase ratio and the prevalence of NAFLD by gender **(A)**, age **(B)**, BMI **(C)**, and race **(D)**. They were adjusted for age, gender, race, hypertension, BMI, T2DM, smoke, WC, LSM, DBP, SBP, CRP, fast glucose, fast insulin, HbA1c, TBIL, ALP, GGT, TC, TG, and SUA. In subgroup analyses, the model was not adjusted for the classified variables.

In the context of our current investigation, we investigated the potential for a non-linear relationship between ALT/AST and NAFLD using smooth curve fits. Subgroup analyses used age, race, gender, and BMI categories. The correlation between ALT/AST and the incidence of NAFLD showed a non-linear pattern (**Table 4**, **Figures 2**, **3**). The inflection point for all participants was 1.359, 1.333 for the BMI over 30 Kg/m^2^ subgroup population, and 1.429 for the cohort under 20 years. In women, the non-linear trend is particularly evident, showing an inverted U-shaped curve with an inflection point of 1.302.

**Table 4:** Threshold effect analysis of ALT/AST on the prevalence of NAFLD in different g genders, ages, and BMI using the two-piecewise linear regression model.

Prevalence of NAFLD	Adjusted OR (95% CI), *P* value
All participants	
Fitting by the standard linear model	6.339 (4.996, 8.044), < 0.0001
Fitting by the two-piecewise linear model	
Inflection point	1.359
ALT/AST < 1.359	13.130 (9.384, 18.371), < 0.0001
ALT/AST > 1.359	1.299 (0.768, 2.197), 0.1812
Log likelihood ratio	<0.001
Women	
Fitting by the standard linear model	6.605 (4.546, 9.598), < 0.0001
Fitting by the two-piecewise linear model	
Inflection point	1.302
ALT/AST < 1.302	19.305 (11.705, 31.838), < 0.0001
ALT/AST > 1.302	0.295 (0.103, 0.844), 0.0228
Log likelihood ratio	<0.001
Age < 20 years	
Fitting by the standard linear model	18.336 (9.078, 37.036), < 0.0001
Fitting by the two-piecewise linear model	
Inflection point	1.429
ALT/AST < 1.429	70.548 (25.649, 194.044), < 0.0001
ALT/AST > 1.429	1.058 (0.223, 5.020), 0.9435
Log likelihood ratio	<0.001
BMI > 30 Kg/m^2^	
Fitting by the standard linear model	3.322 (2.297, 4.805), < 0.0001
Fitting by the two-piecewise linear model	
Inflection point	1.333
ALT/AST < 1.333	6.742 (3.920, 11.598), < 0.0001
ALT/AST > 1.333	0.972 (0.469, 2.016), 0.9396
Log likelihood ratio	<0.001

Age, gender, race, hypertension, BMI, T2DM, smoke, physical activity level, WC, LSM, DBP, SBP, CRP, fast glucose, fast insulin, HbA1c, TBIL, ALP, GGT, TC, TG, and SUA were adjusted. In the subgroup analysis for gender, the model was not adjusted for gender; in the subgroup analysis for age, the model was not adjusted for age; and in the subgroup analysis for BMI, the model was not adjusted for BMI.

### 3.6 ALT/AST as a predictor of NAFLD: ROC analysis

**Figure 4:**
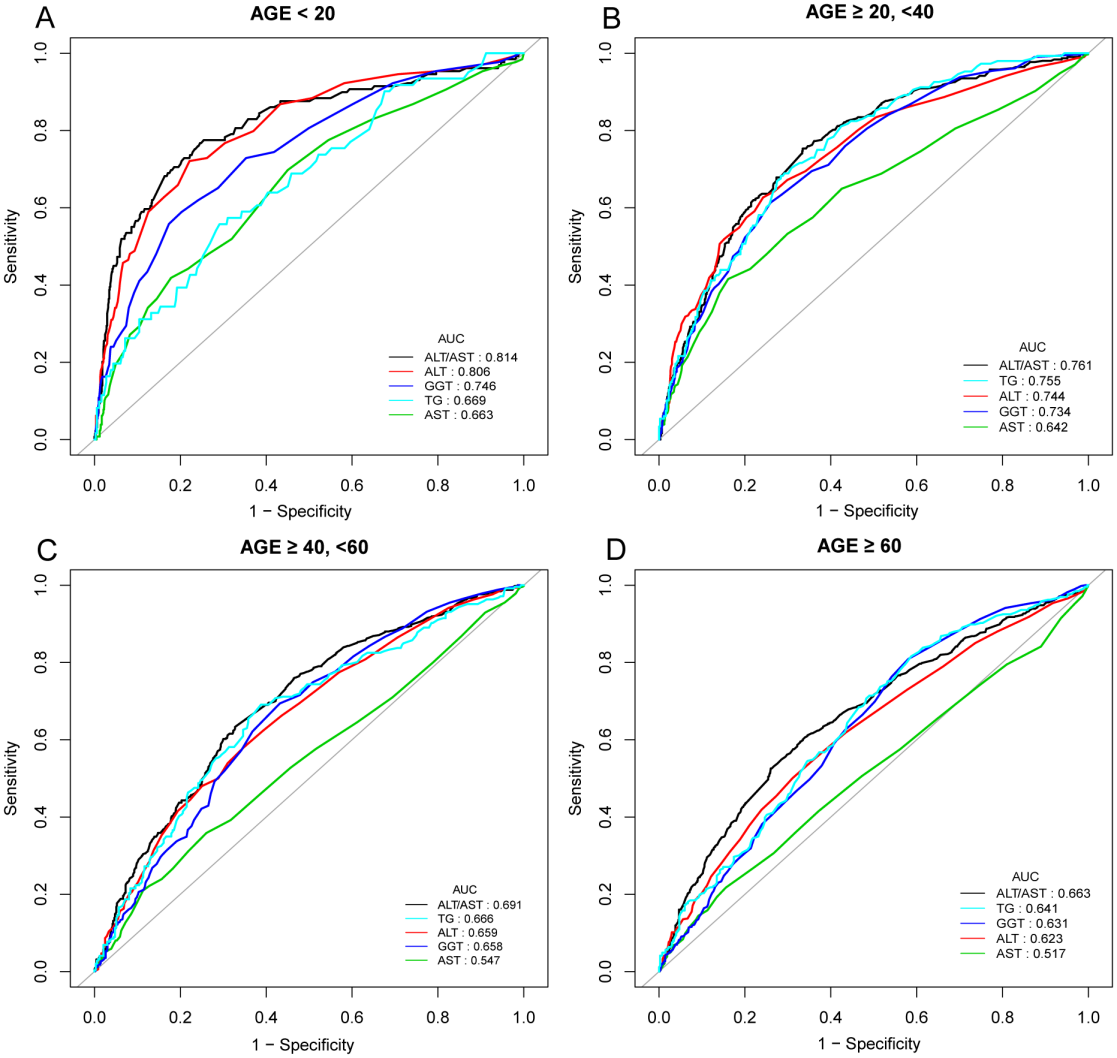
ROC curves for ALT/AST, compared to ALT, AST, GGT, and TG for NAFLD onset in various age groups: AGE < 20 years **(A)**, AGE ≥ 20, <40 years **(B)**, AGE ≥ 40, <60 years **(C)**, and AGE ≥ 60 years **(D)**. As determined by AUC, the predictive value for ALT/AST is more significant than other factors.

In previous research, ROC curve analysis identified ALT, AST, GGT, and TG as significant predictors of NAFLD. Compared to these predictors, the ROC for ALT/AST has been displayed (**Figure 4**, **Supplementary Figures 1**, **2**, **Supplementary Table 2**). The ROC analysis’s area under the curve (AUC) for ALT/AST was 0.7253 (95% CI: 0.7099, 0.7407), substantially more significant compared to that of ALT, AST, GGT, and TG (*P* < 0.001) (**Supplementary Table 2**). The sensitivity and specificity of ALT/AST for NAFLD were estimated to be 70.03% and 65.45%, respectively. We also performed a gender subgroup analysis and found that the AUC for ALT/AST in the ROC analysis was higher than that of the other indicators(**Supplementary Figure 2**, **Supplementary Table 2**). The AUC for ALT/AST was 0.7248 (95% CI: 0.7028, 0.7468) in men and 0.7154 (95% CI: 0.6932, 0.7375) in women. We performed a subgroup analysis based on age (**Figure 4**, **Supplementary Table 2**). We found that the ROC analysis’s AUC for ALT/AST was higher than that of the other indicators.

## 4 Discussion

According to research currently available, the majority of people with NAFLD do not exhibit any particular symptoms or occult symptoms; in fact, 25% of patients may experience inflammation, hepatocyte damage, liver fibrosis, liver cirrhosis, or even liver failure as a result of chronic liver fat accumulation (33). Moreover, those with fatty liver disease have an increased risk of developing cardiovascular and cerebrovascular diseases (28). Thus, it is of great clinical significance to identify NAFLD early and take active treatment measures to interfere with the progress of NAFLD.

Through a sizable nationwide cross-sectional study in the US, this research concentrated on the association between ALT/AST and the risk of NAFLD, the degree of hepatic steatosis, and liver fibrosis. According to our study, high ALT/AST ratios may be strongly linked in the general population to the onset of NAFLD, the severity of hepatic steatosis, and an increased risk of liver fibrosis. After adjusting for possible confounders, each unit increase in ALT/AST was associated with a 3.648-fold rise in the incidence of NAFLD. Moreover, additional subgroup analysis based on age, gender, and BMI stratification revealed a statistically significant positive correlation between each subgroup’s incidence of NAFLD and increased ALT/AST ratios. It is worth noting that there is also a non-linear correlation and saturation effect between the increase in ALT/AST ratio and the occurrence of NAFLD in the above subgroups. In the subgroup analysis based on gender, we found that the ALT/AST ratio and the incidence of NAFLD showed a nonlinear relationship for both men and women. This was particularly prominent in women, presenting an inverted U-shaped curve with an inflection point of 1.302. We speculate that it may be related to the hormone level of women, especially the estrogen level. Therefore, it is recommended that the role of ALT/AST in different sexes be considered when assessing the risk of NAFLD. Besides, ROC analysis in this study indicated that the ALT/AST ratio was superior to ALT or AST alone in detecting NAFLD. In the future, the ALT/AST ratio might help estimate the risk of NAFLD in the general population, given its ease of calculation.

Previous studies of the ALT/AST ratio have focused on the correlation between the ALT/AST ratio and cardiometabolic disease risk, muscle mass in patients with T2DM, and gastrointestinal tumors (21, 34, 35). However, recent studies have found that ALT/AST value is a marker of systemic inflammation and oxidative stress; high ALT/AST ratio indicates fatty liver and insulin resistance, and it is related to metabolic syndrome and can reflect liver injury and advanced inflammatory process. The serum ALT/AST value can better reflect the fat accumulation in the liver than the traditional liver enzyme index (36, 37).

The relationship between the ALT/AST ratio and NAFLD has not been extensively studied. The first cross-sectional study of 10,724 healthy subjects conducted by Lee et al. in South Korea showed that high ALT/AST ratio, high BMI, and diabetes were independent risk factors of NAFLD (38). In patients with chronic hepatitis C virus infection, particularly those who are not genotype 3, the ALT/AST ratio may be a significant risk factor for hepatic steatosis, according to later research conducted in Taiwan on 1354 individuals over 20 years old (37). The ALT/AST ratio was later shown to be an independent risk factor for NAFLD by Shi et al. in a cross-sectional case-control study of 6,926 participants who underwent health assessments (39). Guan et al. conducted a retrospective analysis of 3959 healthy women. They found that high ALT/AST, TG/HDLC, BMI, and UA were independent risk factors for the development and severity of NAFLD. ALT/AST had the strongest correlation with NAFLD (40). During a 5-year follow-up, Zou et al.’s recent longitudinal cohort research of 12,127 Chinese participants who were not obese discovered that the onset of NAFLD was independently linked with a higher ALT/AST ratio. This independent connection was more significant in individuals with hypertension, hyperlipidemia, hyperglycemia, and males (17). These studies were consistent with our research, showing a robust association between ALT/AST and NAFLD. In addition, our gender subgroup analysis provided data suggesting that women with high ALT/AST ratios are significantly more likely to develop NAFLD than men. Moreover, compared to other conventional liver enzyme markers, our research indicates that ALT/AST has a substantially higher AUC value for diagnosing NAFLD. Accordingly, the ALT/AST ratio is associated with NAFLD in the general population, and it may be a useful diagnostic indicator of NAFLD, according to an increasing amount of clinical research (41).

However, previous studies have focused on Asian populations. Our study is the first to examine the association between an elevated ALT/AST ratio and the risk of NAFLD in a large sample of the general American population. Based on the above findings, the ALT/AST ratio may be a valuable indicator of liver inflammation when diagnosing NAFLD in Asian and American general populations.

According to current research, there may be three reasons for the increase in AST/ALT levels associated with NAFLD (1): With the increase in the proportion of fatty tissue in the liver, the burden on normal hepatocytes is aggravated and further destroyed, and a large amount of ALT in the cytoplasm release into the serum, leading to the increase in ALT/AST levels; (2) ALT/AST is related to indocyanine green clearance rate, while indocyanine green clearance rate is related to hepatic blood flow. The decrease in hepatic blood flow in the case of a large proportion of fatty tissue will lead to an increase in ALT/AST; (3) the degree of damage to liver cells in patients with NAFLD is usually mild, and the damage site is generally limited to the liver cell membrane. The physiological structure of mitochondria is intact, so the amount of AST released into the serum is low, and the ALT/AST value will increase accordingly (42, 43). Further research is needed to clarify the mechanism underlying the link between the two.

In our study, subgroup analysis stratified by age showed that an elevated ALT/AST ratio was associated with a higher risk of NAFLD in children and adolescents younger than 20 years compared with participants in other age groups, with an AUC of 0.811, an optimal threshold of 0.865, a sensitivity of 0.767 and a specificity of 0.754. A previous community-based study by Lu et al. in Taiwan included 1222 children and adolescents aged 10-19. The investigation indicated that according to ROC curve analysis, the risk score for fatty liver based on the ALT/AST ratio was more significant than 0.981, and the AUC was 0.756, suggesting that the ALT/AST ratio may be a strong indicator of liver steatosis in children and adolescents (44). Although there have been cases of hepatic steatosis in neonates, little is known about the condition in preadolescent children. NAFLD’s development and subsequent natural history in childhood and adolescence is unknown (45, 46). The majority of children with NAFLD often present with clinical symptoms between the ages of 10 and 13 (47, 48). It was reported by Takahashi et al. that NAFLD in children showed different histopathological characteristics from those in adults. In this research, they found that fatty degeneration in children was more severe than that in adults. The AST and ALT values of children with confirmed NASH reflect the histopathological severity of NAFLD (49). Lifestyle factors, including the habit of eating snacks and lack of physical exercise, are related to the high risk of NAFLD among adolescents in high-income countries. The difference in NAFLD prevalence among different income countries needs further investigation (50). Therefore, clinicians should monitor and manage the onset and progression of NAFLD using the ALT/AST ratio in early childhood and adolescence to improve prognosis and prevent progression through lifestyle changes or medication.

Another interesting finding in our study was that ALT/AST was associated with significant liver fibrosis and advanced liver fibrosis in the general population as well as in men. However, we do not find an association with advanced liver fibrosis or cirrhosis in females. These findings may be explained by the protective effect of estrogen on fibrogenesis. The average age of women in this study was less than 50 years old, which was younger than the average age of menopause. Estrogen inhibits stellate cell activation and fiber formation. Therefore, sex and reproductive status may affect the degree of fibrosis in patients with nonalcoholic steatohepatitis (NASH). Compared with premenopausal women, men have a higher risk of severe liver fibrosis, while postmenopausal women have a similar degree of liver fibrosis to men (51).

The main advantages of our research are listed below. The primary significance of this study is the large sample size of the general population, representing the entire country of the United States, and the accurate assessment of liver steatosis and fibrosis using TE. The population can also be studied in subgroups due to the large sample size. Secondly, this study shows for the first time that the ALT/AST ratio can independently predict liver fibrosis. However, our study has some limitations. First, we cannot establish the causal relationship based on this cross-sectional study; therefore, further prospective cohort studies should be conducted to confirm the causal relationship. Second, although liver biopsy is the most accurate approach to diagnosing NAFLD, histological confirmation was lacking in this study. Using TE with CAP ≥ 288dB/m instead of liver biopsy to diagnose NAFLD may be biased. Several variables, including BMI, diabetes, and the use of M/XL probes, may influence CAP levels. Third, even after adjusting for many relevant covariates, there is potential residual confounding by other factors, such as hyperlipidemia, hypertension and diabetes medication, and unrecorded covariates. Fourth, there may be a bias in the selection process of the study, as some people who have not completed or have only partially completed the TE will not be included. In addition, the study could not obtain new data because it was a secondary analysis. These issues should be the focus of future investigation.

## 5 Conclusion

In summary, a higher ALT/AST ratio was independently associated with a significantly higher risk of NAFLD and liver fibrosis within American cohorts. This link is robust among females, children, and adolescents. ALT/AST ratio can be used as a simple and effective noninvasive biomarker to identify individuals with high risk of NAFLD, which is helpful for the early detection of this prevalent liver disease.

## Data Availability

Publicly available datasets were analyzed in this study. This data can be found here: The datasets collected and analyzed during the current study are available on the NHANES website (http://www.cdc.gov/nchs/nhanes.htm).
