# Supplementary material for: Elevated ALT/AST ratio as a marker for NAFLD risk and severity: insights from a cross-sectional analysis in the United States
Source: Front Endocrinol (Lausanne). 2024 Aug 26;15:1457598. doi: 10.3389/fendo.2024.1457598 (PMC11381241; doi:10.3389/fendo.2024.1457598)
Supplement: Supplementary file 1 [file DataSheet1.docx]

Supplementary Material

**1 Supplementary Figures and Tables**

- 1. **Supplementary Figures**

**
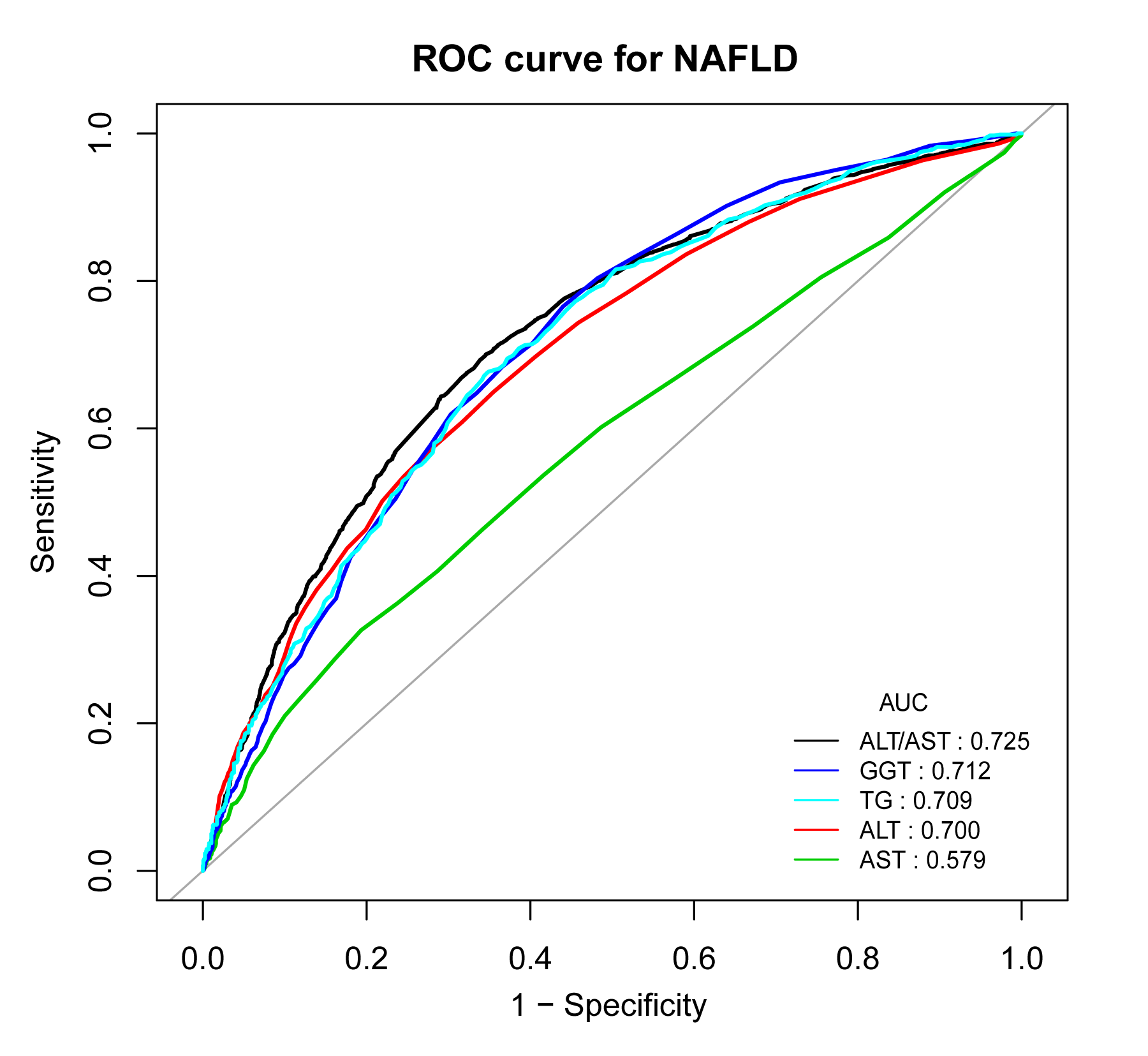
**

**Supplementary Figure 1.** ROC curves for ALT/AST, compared to ALT, AST, GGT, and TG for NAFLD onset. As determined by AUC, the predictive value for ALT/AST is more significant than other factors.


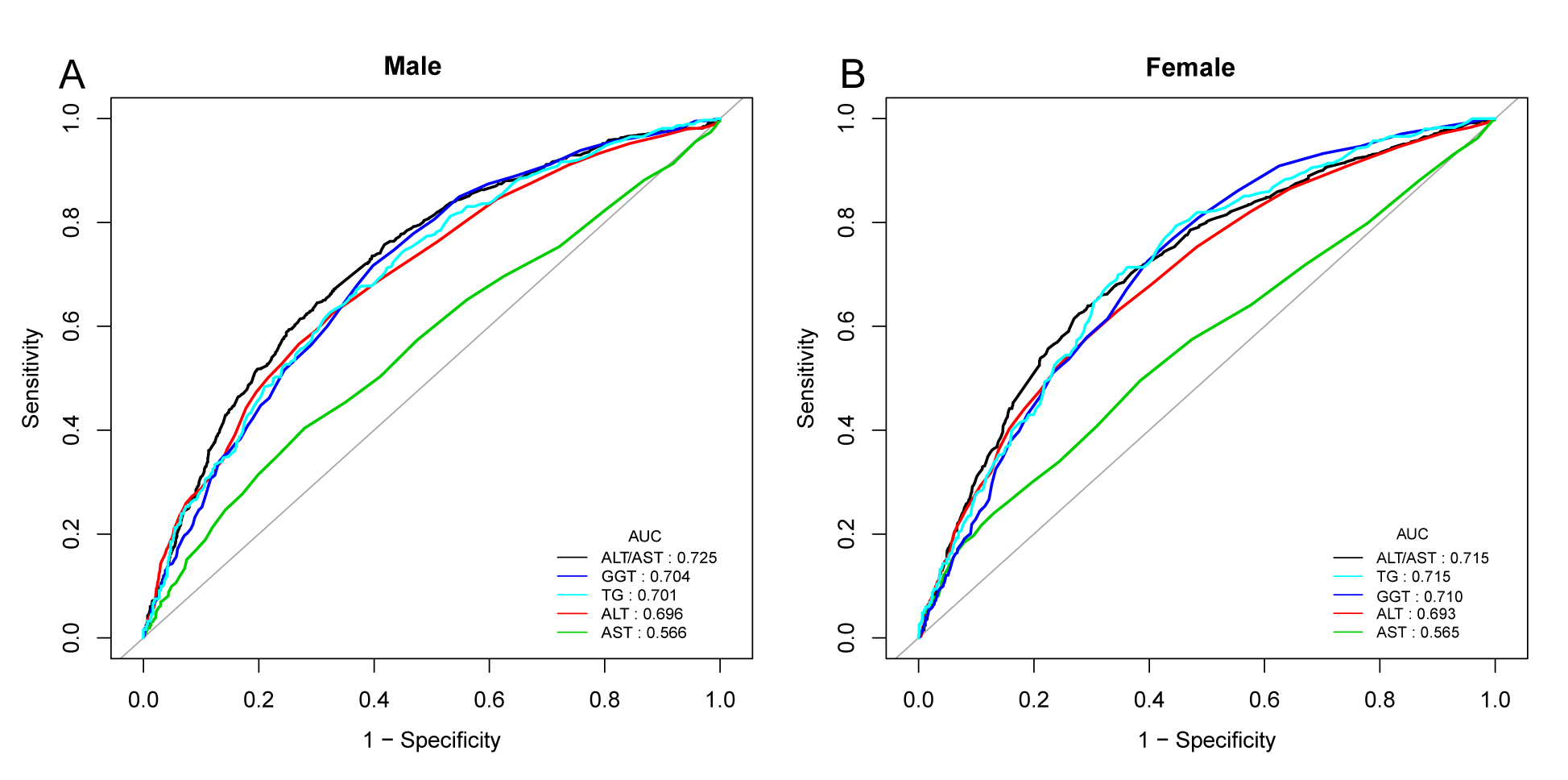
**Supplementary Figure 2.** ROC curves for ALT/AST, compared to ALT, AST, GGT, and TG for NAFLD onset among males (A) and females (B). The predictive value for ALT/AST is more significant than other factors, as determined by its AUC.

- 1. **Supplementary Tables**

**Supplemental Table 1.** Correlation between ALT/AST and hepatic steatosis based on CAP value.

|  | **Model 1 β (95% CI), *P* value** | **Model 2 β (95% CI), *P* value** | **Model 3 β (95% CI), *P* value** |
| --- | --- | --- | --- |
| **ALT/AST**  (per SD increase) | 70.570 (65.916, 75.224)  < 0.001 | 67.137 (62.499, 71.774)  < 0.001 | 31.551 (26.806, 36.296)  < 0.001 |
| Q1 (0.167-0.707) | Reference | Reference | Reference |
| Q2 (0.708-0.881) | 17.216 (12.537, 21.894)  < 0.001 | 11.602 (7.080, 16.123)  < 0.001 | 2.045 (-2.006, 6.096)  < 0.001 |
| Q3 (0.882-1.130) | 38.778 (34.116, 43.440)  < 0.001 | 30.839 (26.279, 35.399)  < 0.001 | 10.032 (5.829, 14.235)  < 0.001 |
| Q4 (1.133-3.314) | 69.208 (64.544, 73.872)  < 0.001 | 62.788 (58.140, 67.436)  < 0.001 | 27.823 (23.222, 32.425)  < 0.001 |
| *P* for trend | < 0.001 | < 0.001 | < 0.001 |
| **Subgroup analysis stratified by sex** | | | |
| Men | 67.626 (61.377, 73.874)  < 0.001 | 65.960 (59.950, 71.971)  < 0.001 | 30.066 (23.639, 36.494)  < 0.001 |
| Women | 74.448 (66.871, 82.024)  < 0.001 | 68.984 (61.595, 76.373)  < 0.001 | 29.812 (22.529, 37.094)  < 0.001 |
| **Subgroup analysis stratified by age** | | | |
| < 20 | 73.353 (64.089, 82.617)  < 0.001 | 73.409 (63.953, 82.865)  < 0.001 | 34.637 (24.179, 45.095)  < 0.001 |
| ≥ 20, <40 | 66.782 (58.983, 74.582)  < 0.001 | 60.762 (52.234, 69.291)  < 0.001 | 25.890 (17.403, 34.377)  < 0.001 |
| ≥ 40, <60 | 64.024 (54.831, 73.217)  < 0.001 | 61.356 (51.497, 71.214)  < 0.001 | 32.165 (23.064, 41.267)  < 0.001 |
| ≥ 60 | 66.682 (55.653, 77.711)  < 0.001 | 62.780 (51.447, 74.114)  < 0.001 | 34.369 (22.844, 45.894)  < 0.001 |
| **Subgroup analysis stratified by BMI** | | | |
| < 25 | 36.269 (27.994, 44.544)  < 0.001 | 23.466 (15.422, 31.510)  < 0.001 | 20.605 (12.090, 29.121)  < 0.001 |
| ≥ 25, <30 | 42.586 (34.727, 50.445)  < 0.001 | 38.422 (30.279, 46.564)  < 0.001 | 34.414 (25.435, 43.392)  < 0.001 |
| ≥ 30 | 46.876 (39.768, 53.984)  < 0.001 | 42.127 (34.397, 49.858)  < 0.001 | 33.025 (24.972, 41.078)  < 0.001 |

Model 1: no covariates were adjusted. Model 2: age, gender, and race were adjusted. Model 3: age, gender, race, hypertension, BMI, T2DM, smoke, physical activity level, WC, LSM, DBP, SBP, CRP, fast glucose, fast insulin, HbA1c, TBIL, ALP, GGT, TC, TG, and SUA were adjusted. In the subgroup analysis for gender, the model was not adjusted for gender; in the subgroup analysis for age, the model was not adjusted for age; in the subgroup analysis for BMI, the model was not adjusted for BMI.

**Supplemental Table 2.** The best threshold, sensitivities, specificities, and area under the curve of each parameter for screening NAFLD in the general population and subgroup analysis for gender, age, and BMI.

|  | **AUC** | **95% CI** | **Best threshold** | **Specificity** | **Sensitivity** |
| --- | --- | --- | --- | --- | --- |
| ALT/AST | 0.7253 | 0.7099-0.7407 | 0.9183 | 0.6545 | 0.7003 |
| ALT | 0.7005* | 0.6846-0.7164 | 17.5000 | 0.6453 | 0.6492 |
| AST | 0.5793* | 0.5614-0.5972 | 23.5000 | 0.8062 | 0.3263 |
| GGT | 0.7123* | 0.6971-0.7275 | 17.5000 | 0.5602 | 0.7653 |
| TG | 0.7092* | 0.6869-0.7316 | 91.5000 | 0.6514 | 0.6768 |
| **Subgroup analysis stratified by sex** | | | | | |
| **Men** | | | | | |
| ALT/AST | 0.7248 | 0.7028-0.7468 | 1.0580 | 0.6988 | 0.6453 |
| ALT | 0.6964* | 0.6735-0.7193 | 21.5000 | 0.6710 | 0.6264 |
| AST | 0.5659* | 0.5403-0.5914 | 23.5000 | 0.7206 | 0.4038 |
| GGT | 0.7042* | 0.6819-0.7264 | 21.5000 | 0.6018 | 0.7170 |
| TG | 0.7006* | 0.6678-0.7334 | 98.5000 | 0.6768 | 0.6263 |
| **Women** |  |  |  |  |  |
| ALT/AST | 0.7154 | 0.6932-0.7375 | 0.9183 | 0.7220 | 0.6241 |
| ALT | 0.6928* | 0.6702-0.7154 | 16.5000 | 0.7076 | 0.5778 |
| AST | 0.5649* | 0.5393-0.5906 | 18.5000 | 0.6158 | 0.4951 |
| GGT | 0.7102* | 0.6871-0.7313 | 16.5000 | 0.6030 | 0.7251 |
| TG | 0.7145* | 0.6837-0.7454 | 87.5000 | 0.6540 | 0.6991 |
| **Subgroup analysis stratified by age** | | | | | |
| **< 20** | | | | | |
| ALT/AST | 0.8114 | 0.7691-0.8596 | 0.8652 | 0.7540 | 0.7674 |
| ALT | 0.8059* | 0.7629-0.8489 | 17.5000 | 0.7786 | 0.7209 |
| AST | 0.6629* | 0.6104-0.7153 | 18.5000 | 0.5498 | 0.6977 |
| GGT | 0.7464* | 0.6992-0.7936 | 17.5000 | 0.7970 | 0.5891 |
| TG | 0.6692* | 0.5957-0.7426 | 77.5000 | 0.7088 | 0.5574 |
| **≥ 20, < 40** |  |  |  |  |  |
| ALT/AST | 0.7609 | 0.7302-0.7916 | 0.9583 | 0.6656 | 0.7532 |
| ALT | 0.7438* | 0.7109-0.7767 | 21.5000 | 0.7578 | 0.6266 |
| AST | 0.6422* | 0.6042-0.6802 | 23.5000 | 0.8389 | 0.4156 |
| GGT | 0.7338* | 0.7022-0.7653 | 22.5000 | 0.7400 | 0.6136 |
| TG | 0.7549* | 0.7119-0.7979 | 92.0000 | 0.7165 | 0.6824 |
| **≥ 40, < 60** | | | | | |
| ALT/AST | 0.6909 | 0.6608-0.7209 | 1.0106 | 0.6725 | 0.6333 |
| ALT | 0.6591* | 0.6282-0.6901 | 20.5000 | 0.6522 | 0.5804 |
| AST | 0.5470* | 0.5138-0.5803 | 22.5000 | 0.7391 | 0.3588 |
| GGT | 0.6583* | 0.6276-0.6890 | 21.5000 | 0.5681 | 0.6941 |
| TG | 0.6657* | 0.6210-0.7104 | 96.5000 | 0.6119 | 0.6911 |
| **≥ 60** |  |  |  |  |  |
| ALT/AST | 0.6632 | 0.6340-0.6924 | 0.9024 | 0.6603 | 0.6061 |
| ALT | 0.6226* | 0.5926-0.6525 | 18.5000 | 0.6888 | 0.5009 |
| AST | 0.5175* | 0.4861-0.5488 | 25.5000 | 0.8444 | 0.2175 |
| GGT | 0.6311* | 0.6020-0.6601 | 16.5000 | 0.4192 | 0.8093 |
| TG | 0.6406* | 0.5984-0.6828 | 78.5000 | 0.4129 | 0.8158 |

* *P* < 0.001, compared with ALT/AST.
